# Evaluation of the Antioxidant Potential of *Salvia miltiorrhiza* Ethanol Extract in a Rat Model of Ischemia-Reperfusion Injury

**DOI:** 10.3390/molecules161210002

**Published:** 2011-12-02

**Authors:** Zengyong Qiao, Jiangwei Ma, Huajin Liu

**Affiliations:** 1 Department of Cardiology, Fengxian Branch of Shanghai 6th People’s Hospital, Shanghai 201400, China; Email: qiaozydrfx63@sohu.com (Z.Q.); liuhjdrfx26@sohu.com (H.L.); 2 Tongji University School of Medicine, Shanghai 200092, China

**Keywords:** *Salvia miltiorrhiza* ethanol extract, rat, oxidative injury

## Abstract

The present study was undertaken to evaluate the protection potential of ethanol extract of *Salvia miltiorrhiza* (SMEE) against oxidative injury in the ischemia-reperfusion (I/R) model of rats *in vivo*. Rats were divided into six groups of 10 rats each. Group I/R model and sham were fed with a standard rat chow, groups SMEE I and SMEE II were fed with a standard rat chow and 400 or 800 mg/kg b.w. ethanol extract for 12 days before the beginning of I/R studies. Positive control group was fed with a standard rat chow and salvianolic acid B (55 mg/kg b.w.) or tanshinone II-A (55 mg/kg b.w.) for 12 days before the beginning of I/R studies. To produce I/R, the left anterior descending artery (LAD) was occluded in anesthetized rats for 15 min, followed by 120 min reperfusion. Infarct sizes were found significantly decreased in SMEE-treated and positive control groups compared to I/R model group. Serum AST, LDH and CK-MB activities were significantly reduced and myocardium Na^+^-K^+^ ATPase, Ca^2+^-Mg^2+^ ATPase activities and antioxidant enzyme activities (SOD, CAT, GSH-Px) were markedly increased in SMEE-treated and salvianolic acid B or tanshinone II-A positive control groups compared to the I/R model group. Pretreatment of *S. miltiorrhiza* ethanol extract and salvianolic acid B or tanshinone II-A dose-dependently reduced significantly myocardium MDA level, ROS and NOS activities and enhanced myocardium GSH level in I/R rats compared to I/R rats model. In conclusion, we clearly demonstrated that *S. miltiorrhiza* ethanol extract pretreatment can decrease oxidative injury in rats subjected to myocardial I/R.

## 1. Introduction

Myocardial ischemia-reperfusion (I/R) injury is known to occur on restoration of coronary flow after a period of myocardial ischemia usually caused by coronary heart disease. Injury of myocardium due to I/R includes cardiac contractile dysfunction, arrhythmias, as well as irreversible myocyte damage *i.e.*, myocardial necrosis [1].

Elevated levels of oxidizing free radicals following reperfusion provide a rationale for the testing of antioxidants in models of myocardial ischemia/reperfusion injury [2]. Reactive oxygen species (ROS) can cause oxidative damage to a variety of cellular components. ROS play an important role in the etiology of myocardial ischemia-reperfusion (I/R) injury [3,4]. During ischemia, the coronary blood supply to the heart is reduced or stopped preventing oxygen, glucose, and fatty acids from reaching the target tissue [5]. Ischemia inactivates oxidative phosphorylation, leading to a loss of adenine nucleotides and cytochrome c, accumulation of free phosphate, fatty acids, and lactic acid, increased cellular calcium, and a decrease in cellular pH [6].

*S. miltiorrhiza*, also known as danshen, is a shade-growing perennial flowering plant that belongs to the family of Labiatae. It is highly valued for its roots in traditional Chinese medicine (TCM), which according to the therapeutic theory of Chinese medicine, has properties of promoting blood circulation, relieving blood stasis, clearing heat from the blood, resolving swelling and tranquillizing the mind. Danshen is often used in decoction preparations, either individually or in combination with other TCM for treating dementia and cardiovascular diseases such as angina pectoris, myocardial infarction, stroke, and hyperlipidemia [7,8,9,10].

In the present study, on the one hand, we evaluated the effect of the ethanol extract of *S. miltiorrhiza* (SMEE) on the preservation of cardiac tissue integrity, measuring the effects on ST segment in the rat electrocardiogram, heart weigh and infarcted area and on serum lactate dehydrogenase (LDH), aspartate transaminase (AST) and creatine phosphokinase-muscle band (CK-MB) activities. On the other hand, we studied the potential effect of SMEE on Ca^2+^ input in cardiac cells, determining its effect on the modulation of myocardial Na^+^-K^+^ ATPase and Ca^2+^-Mg^2+^ ATPase. Finally, we evaluated the impact of SMEE on antioxidant system, measuring its effects on lipid peroxidation product malondialdehyde (MDA), endogenous antioxidant glutathione (GSH), reactive oxygen species (ROS), nitric oxide synthase (NOS), superoxide dismutase (SOD), catalase (CAT), and glutathione peroxidase (GSH-Px) activities.

## 2. Results

Variations of the ST segment in the rat electrocardiogram between groups wasn’t significant before ligation. Ten and twenty minutes after ligation, I/R-induced elevation of the ST segment in the electrocardiogram was observed (P < 0.01) in I/R model group compared to sham group. Pretreatment of *S.* *miltiorrhiza* ethanol extract significantly (P < 0.05; P < 0.01) reversed I/R-induced elevation of the ST segment in the rat electrocardiogram in a dose-dependent manner (Table 1). Likewise, pretreatment of salvianolic acid B or tanshinone IIA significantly (P < 0.01) reversed I/R-induced elevation of the ST segment in the rat electrocardiogram (Table 1).

**Table 1:** Effect of *S. miltiorrhiza* ethanol extract on the ST segment in the rat electrocardiogram.

Group	Before ligation (mV)	After ligation (mV)	
		10 min	20 min
IR model	0.207 ± 0.006	0.381 ± 0.019 **	0.413 ± 0.018 **
Sham	0.212 ± 0.005	0.218 ± 0.018	0.253 ± 0.016
SMEE I	0.209 ± 0.009	0.321 ± 0.022 ^#^	0.372 ± 0.018 ^#^
SMEE II	0.208 ± 0.008	0.261 ± 0.021 ^##^	0.298 ± 0.017 ^##^
Salvianolic acid B	0.209 ± 0.006	0.254 ± 0.014 ^##^	0.287 ± 0.017 ^##^
Tanshinone IIA	0.210 ± 0.007	0.251 ± 0.016 ^##^	0.279 ± 0.015 ^##^

** *P* < 0.01; compared with sham group; ^#^ *P* < 0.05, ^##^ *P* < 0.01, compared with I/R model group.

There was no marked difference (P > 0.05) in rats’ weight of area at risk between groups. There was no marked difference (P > 0.05) in rats’ cardiac ventricle weight between groups. Compared with I/R model group, the weight of infarcted myocardium and percentage of infarcted area in SMEE I and II groups were significantly decreased (P < 0.05; P < 0.01) in a dose-dependent manner. In addition, the weight of infarcted myocardium and infarcted area in salvianolic acid B or tanshinone IIA groups were decreased, and significant statistical difference (P < 0.01) was found between I/R model group and salvianolic acid B or tanshinone IIA groups (Table 2).

**Table 2:** Effect of *S. miltiorrhiza* ethanol extract on the cardiac ventricle weight, the weight of infarcted myocardium and percentage of infarcted area.

Group	Cardiac ventricle weight (g)	Area at risk (g)	Weight of infarcted myocardium (g)	Percentage of infarcted area (IS/LV) (%)
IR model	0.593 ± 0.024	0.289 ± 0.015	0.07 ± 0.007	12.18 ± 1.15
Sham	0.602 ± 0.031	0.000 ± 0.000	0.000 ± 0.000	0.000 ± 0.000
SMEE I	0.608 ± 0.033	0.124 ± 0.013	0.059 ± 0.005 ^#^	9.84 ± 1.01 ^#^
SMEE II	0.608 ± 0.029	0.126 ± 0.015	0.048 ± 0.005 ^##^	7.87 ± 0.89 ^##^
salvianolic acid B	0.599 ± 0.031	0.127 ± 0.012	0.051 ± 0.005 ^##^	8.42 ± 0.64 ^##^
tanshinone IIA	0.605 ± 0.03	0.126 ± 0.013	0.053 ± 0.004 ^##^	8.65 ± 0.53 ^##^

^#^ *P* < 0.05, ^##^ *P* < 0.01, compared with I/R model group; IS: infarct size; LV: left ventricle.

Table 3 shows the effects of the *S. miltiorrhiza* ethanol extract and salvianolic acid B or tanshinone IIA on rat serum AST, LDH and CK-MB activities. I/R operation significantly (P < 0.01) induced the elevation of serum AST, LDH and CK-MB activities in rats compared to sham group. Both *Salvia miltiorrhiza* ethanol extract and salvianolic acid B or tanshinone IIA exerted significant (P < 0.05; P < 0.01) decrease in the activity of these enzymes.

Table 4 shows the effects of the *S. miltiorrhiza* ethanol extract and salvianolic acid B or tanshinone IIA on myocardium Na^+^-K^+^ ATPase and Ca^2+^-Mg^2+^ ATPase activities in experimental rats. Myocardium Na^+^-K^+^ ATPase and Ca^2+^-Mg^2+^ ATPase activities in I/R model group were significantly (P < 0.01) lower than those in sham group. The myocardium Na^+^-K^+^ ATPase and Ca^2+^-Mg^2+^ ATPase activities dose-dependently significantly (P < 0.01) increased in SMEE-treated animals when compared with the I/R model group. Likewise, the activity of myocardium Na^+^-K^+^ ATPase and Ca^2+^-Mg^2+^ ATPase activities significantly (P < 0.01) increased in salvianolic acid B or tanshinone IIA-treated animals when compared with the I/R model group.

**Table 3:** Effect of *S. miltiorrhiza* ethanol extract on the serum AST, LDH and CK-MB activities.

Group	AST (U/L)	LDH (U/L)	CK-MB (U/L)
IR model	94.12 ± 5.43 **	6023.1 ± 308.54 **	285.32 ± 26.13 **
Sham	43.16 ± 2.97	3068.32 ± 185.72	121.53 ± 9.54
SMEE I	70.32 ± 6.04 ^##^	5107.44 ± 496.11 ^#^	205.57 ± 17.05 ^##^
SMEE II	53.29 ± 3.77 ^##^	4216.35 ± 254.03 ^##^	142.04 ± 11.64 ^##^
Salvianolic acid B	49.76 ± 3.07 ^##^	3597.54 ± 185.39 ^##^	132.49 ± 8.59 ^##^
Tanshinone IIA	50.63 ± 4.11 ^##^	3508.38 ± 208.31 ^##^	128.06 ± 10.14 ^##^

**P < 0.01; compared with sham group; ^#^*P* < 0.05, ^##^*P* < 0.01, compared with I/R model group.

**Table 4:** Effect of *S. miltiorrhiza* ethanol extract on the myocardium Na^+^-K^+^ ATPase and Ca^2+^-Mg^2+^ ATPase activities.

Group	Na^+^-K^+^ ATPase/(μmol∙Pi∙mg^−1^∙h^−1^)	Ca^2+^-Mg^2+^ ATPase/(μmol Pi∙mg^−1^∙h^−1^)
IR model	2.73 ± 0.15 **	4.05 ± 0.31 **
Sham	6.82 ± 0.34	7.91 ± 0.45
SMEE I	4.62 ± 0.33 ^##^	5.87 ± 0.35 ^##^
SMEE II	6.03 ± 0.41 ^##^	7.68 ± 0.53 ^##^
Salvianolic acid B	7.58 ± 0.38 ^##^	8.99 ± 0.44 ^##^
Tanshinone IIA	7.06 ± 0.29 ^##^	9.53 ± 0.51 ^##^

** *P* < 0.01; compared with sham group; ^##^ *P* < 0.01, compared with I/R model group.

As shown in Table 5, a marked (P < 0.01) increase in myocardium MDA level, ROS, NOS activities and decrease in myocardium GSH level were observed in I/R model group when compared with the sham animals.

**Table 5:** Effect of *S. miltiorrhiza* ethanol extract on the myocardium MDA, GSH level, and ROS, NOS activities.

Group	MDA (nmol/g prot)	GSH (μmol/g prot)	ROS (U/mg prot)	NOS (U/mg prot)
IR model	7.85 ± 0.54 **	6.83 ± 0.62 **	97.25 ± 6.39 **	4.06 ± 0.27 **
Sham	4.01 ± 0.36	14.29 ± 1.04	39.15 ± 3.14	1.06 ± 0.13
SMEE I	6.77 ± 0.52 ^#^	9.31 ± 1.09 ^##^	70.13 ± 5.99 ^#^	3.19 ± 0.18 ^#^
SMEE II	5.53 ± 0.38 ^##^	14.82 ± 11.54 ^##^	46.12 ± 3.72 ^##^	1.52 ± 0.17 ^##^
Salvianolic acid B	4.29 ± 0.32 ^##^	19.43 ± 1.33 ^##^	30.76 ± 2.75 ^##^	1.26 ± 0.11 ^##^
Tanshinone IIA	5.08 ± 0.29 ^##^	18.38 ± 1.75 ^##^	33.14 ± 2.41 ^##^	1.22 ± 0.09 ^##^

**P < 0.01; compared with sham group; ^#^*P* < 0.05, ^##^*P* < 0.01, compared with I/R model group.

Pretreatment of *S. miltiorrhiza* ethanol extract dose-dependently significantly (P < 0.05; P < 0.01) reduced myocardium MDA level, ROS, NOS activities and enhanced myocardium GSH level in I/R rats compared to I/R model group. In addition, Pretreatment of salvianolic acid B or tanshinone IIA also significantly (P < 0.01) reduced myocardium MDA level, ROS, NOS activities and enhanced myocardium GSH level in I/R rats compared to I/R model group.

As shown in Table 6, a marked (P < 0.01) decrease in myocardium SOD, CAT, and GSH-Px activities were observed in I/R model group when compared with the sham animals. In contrast to the findings in I/R model group, there was a significant (P < 0.05; P < 0.01) increase in the myocardium SOD, CAT, and GSH-Px activities in I/R rats when the ethanol extract was pre-administered into the rats. Pretreatment of salvianolic acid B or tanshinone IIA enhanced significantly (P < 0.01) myocardium SOD, CAT, and GSH-Px activities when compared with I/R model group.

**Table 6:** Effect of *S. miltiorrhiza* ethanol extract on the myocardium SOD, CAT and GSH-Px activities.

Group	SOD (U/mg prot)	CAT (U/mg prot)	GSH-Px (U/mg prot)
IR model	118.3 ± 8.54 **	13.14 ± 1.09 **	10.19 ± 1.09 **
Sham	228.3 ± 12.64	21.82 ± 1.42	21.86 ± 1.49
SMEE I	163.2 ± 11.37 ^##^	17.25 ± 1.27 ^##^	16.03 ± 1.22 ^##^
SMEE II	203.1 ± 18.21 ^##^	20.42 ± 1.85 ^##^	22.71 ± 1.98 ^##^
Salvianolic acid B	227.07 ± 17.04 ^##^	23.04 ± 1.93 ^##^	28.04 ± 1.06 ^##^
Tanshinone IIA	268.4 ± 15.33 ^##^	22.42 ± 1.37 ^##^	26.39 ± 1.44 ^##^

** *P* < 0.01; compared with sham group; ^##^ *P* < 0.01, compared with I/R model group.

## 3. Discussion

In the present study, we have demonstrated that *S. miltiorrhiza* ethanol extract effectively decreased oxidative injury and reduced myocardial infarct size in rat myocardial ischemia and reperfusion model *in vivo*. It has been reported that ischemia and reperfusion leads to several injurious responses in microcirculation, such as an enhanced oxygen free radical production from endothelial cells [11], increased expression levels of CD11b/CD18 in leukocytes and ICAM-1 in endothelial cells [12,13].

The magnitude of damage due to myocardial ischemia may be assayed based on the ST segment elevation. A relationship exists between the extent of ST segment displacement and the size of the infarction. The correlation between ST segment elevation and biochemical enzyme parameters are a matter of debate. There is no conclusive evidence available to prove the efficiency of ECG changes in the diagnosis of acute myocardial infarction [14].

In the present study, I/R-induced elevation of the ST segment in the electrocardiogram was observed in I/R rats model compared to sham group. We confirmed that reperfusion after ischemia caused increase of the weight of infarcted myocardium and percentage of infarcted area which resulted in cardiac contractile dysfunction and ischemic damages in ischemia and reperfusion model *in vivo*. Pretreatment with *Salvia miltiorrhiza* ethanol extract could reverse I/R-induced elevation of the ST segment in the rats’ electrocardiogram and reduce the weight of infarcted myocardium and percentage of infarcted area.

The integrity of cardiac tissue is important in drug biotransformation and metabolism and is highly associated with the level of serum CK, CK-MB and AST. In situations of oxidative damage there is high activity of these enzymes in the blood, in this case the administration of antioxidant vitamin such as vitamin A may ameliorate tissue dysfunction since antioxidant vitamins are known to improve tissue integrity. The present study reveals that myocardium I/R operation causes a marked increase of the serum CK-MB, LDH and AST activities. We observed that pretreatment with SMEE resulted in a significant decrease in serum activity of LDH, AST and CK-MB, showing this extract contributed to preserve cardiac tissue integrity of rats.

The reduction of Na^+^-K^+^-ATPase activity has been shown to enhance myocardial contractility due to increased intracellular Ca^2+^ concentration. Decreased myocardial ATPase activities and intracellular and intramitochondrial Ca^2^^+^ overload may induce myocardial damage such as ischemic cardiac dysfunction, arrhythmias, *etc.* [15].

The current study showed that pre-administration of *S. miltiorrhiza* ethanol extract enhanced Na^+^-K^+^-ATPase and Ca^2+^- Mg^2+^-ATPase activities of I/R rat heart. The increased activities of Na^+^-K^+^-ATPase and Ca^2+^-Mg^2+^-ATPase in the hearts of the IR rats pre-treated with *S. miltiorrhiza* ethanol extract suggest that one of the mechanisms whereby *S. miltiorrhiza* ethanol extract prevents development of I/R-induced cardiac dysfunction may be attributed to enhancement of activities of cardiac Na^+^-K^+^-ATPase and Ca^2+^-Mg^2+^-ATPase.

Oxidative stress is a well established etiopathogenic factor of ischemic heart disease and its consequences. It is now well known that a burst of oxygen free radicals (OFR) occurs immediately after restitution of blood flow to a previously ischemic myocardium [16]. Free radical-mediated injury has been proposed to be one of the major components involved in the pathophysiological alterations observed during ischemia and reperfusion. Generation of reactive oxygen species immediately upon reperfusion has been documented in experimental conditions, as well as in patients with acute myocardial infarction undergoing thrombolysis, coronary angioplasty or open heart surgery [17]. In the heart, reactive oxygen intermediates such as the superoxide anion and the hydroxyl radical, which are formed during reperfusion or reoxygenation of the ischemic tissue, may be responsible for the induction of electrophysiological and biochemical disturbances [18,19,20].

In the present study, pre-administration of *S. miltiorrhiza* ethanol extract caused significant rise in the myocardium antioxidants (SOD, GSH and CAT), and decrease in the myocardium MDA level, ROS, and NOS activities in SMEE-treatment groups. Similar type of observation was seen in salvianolic acid B or tanshinone IIA groups. In the present study, I/R injury was associated with oxidative stress, as evidenced by enhanced lipid peroxidation and deterioration of myocardial endogenous antioxidant status (SOD, GSH and CAT). Similar observations were made earlier in other studies [21,22]. The present study also demonstrated that free radical injury begins during the ischemic phase itself, and as a consequence, if an antioxidant is to be effective, it should be administered before the onset of ischemia. Thus pretreatment with *S. miltiorrhiza* ethanol extract gave better protection against the oxidative stress associated with I/R.

## 4. Experimental

### 4.1. Plant Material

Roots of *S. miltiorrhiza* were obtained from a herb shop in Shanghai, China, in June 2010. The plant material was identified at the department of Traditional Chinese Medicine (Shanghai, China) where a voucher specimen (No. 20100614) was deposited. The root was dried at room temperature and reduced to a powder. Salvianolic acid B and tanshinone II-A were purchased from Shanghai Jiahe Biotechnology Ltd. Their purity was 98%. SMEE was prepared in our laboratory. In brief, roots of *S. miltiorrhiza* (300 g) were extracted with absolute ethyl alcohol (1,200 mL) by triple percolation for 70 min. Re-extraction were done by absolute ethyl alcohol and then the extract was evaporated under vacuum at 60 °C to obtain a brown cream (18 g). The obtained extract was kept at 4 °C. Cary 4000 ultraviolet-visible spectrophotometer [23] showed that salvianolic acid B, tanshinone II-A or 3,4-dihydoxyphenyllactic acid (danshensu) were 16.8%, 0.84%, 6.2%, respectively. Standard samples of salvianolic acid B (HPLC > 98%), tanshinone II-A (HPLC > 98%) or 3,4-dihydoxyphenyllactic acid (danshensu) (HPLC > 98%) were purchased from Shanghai HuiCheng Biotechnology Ltd.

### 4.2. Experimental Procedure

Wistar rats of either sex (220–280 g), 2 months of age, were obtained from the Institute's Experimental Animal Facility of Fengxian Branch of Shanghai 6th People’s Hospital and were kept at 25 °C + 5 °C in a well ventilated animal house under 12 hours light and dark cycle, maintained in compliance with the Guidelines for Animal Experimentation of Fengxian Branch of Shanghai 6th People’s Hospital. Rats, divided into six groups (10 in each group), and fed either with the SMEE in two doses (400 mg/kg b.w., 800 mg/kg b.w.) or with salvianolic acid B (55 mg/kg b.w.; positive control) or tanshinone II-A (55 mg/kg b.w.; positive control), or with vehicle (distilled water), by gavage once a day for 12 days, along with standard rat chow or water *ad libitum*. There was no significant difference in body weight of the treated rats, when compared with control, either at the beginning or at the end of the study period. The treatment schedule did not cause any change in food and water intake pattern.

### 4.3. Surgical Preparation of Animals

At the end of 12 days, rats of all experimental groups were anaesthetized intraperitoneally with pentobarbitone sodium (60 mg/kg). The neck was opened with a ventral midline incision, and a tracheotomy was performed, the rats were ventilated with room air from a positive pressure ventilator (Crompton Parkinson Ltd., England) using compressed air at a rate of 50 strokes/min and a tidal volume of 15 mL/kg. A left thoracotomy was performed and the pericardium was opened to expose the heart. The left arterial descending coronary artery (LAD) was ligated 2 mm from its origin by a 5-0 silk suture with a traumatic needle and ends of this ligature were passed through a small vinyl tube to form a snare. Myocardial ischemia was induced by one stage occlusion of the LAD. Electrocardiographic leads were attached subcutaneous electrodes to monitor limb lead II. The animals then underwent 15 min of ischemia. Then the myocardium was reperfused by releasing the snare gently for a period of 2 hours. The sham control animals were subjected to the entire surgical procedure described above, except the introduction of LAD ligation and release. Electrocardiogram (ECG) (model VS4) was continuously monitored throughout the experiment.

### 4.4. Measurement of Myocardial Infarct Size

To determine the infarct size, at the end of 2 hours reperfusion period, the ligature around the LAD artery re-tied and the heart slowly perfused with 2–3 mL of saline solution containing 0.25% Evans blue dye (w/v) via the side arm of the aortic cannula. The hearts freezed, and then the ventricles of the frozen hearts sliced transversely in a plane perpendicular to the apico-basal axis into 2 mm thick sections. The slices incubated with 1% (w/v) triphenyltetrazolium chloride (TTC) solution in phosphate buffer for 15 min at 37 °C to dye the noninfarcted region [24,25]. This procedure resulted in the normally perfused tissue being stained blue, noninfarcted, nonperfused tissue stained brick red, infarcted tissue remaining unstained and appeared pale [26]. Blood serum samples or myocardial tissues from the area at risk were collected to measure the biochemical indexes.

### 4.5. Enzymes Assays

Serum LDH, AST and CK-MB were determined by using standard kits from BioSystems S.A. (Barcelona, Spain). Na^+^-K^+^-ATPase and Ca^2+^-Mg^2+^-ATPase activities were assayed by spectrophotometrically measuring the amount of inorganic phosphate liberated following incubation of the tissue extract with disodium ATP (Sigma, England) as in previous studies [27,28]. The method employed in the assay of SOD activity was that of Winterbourn *et al.* [29] and is based on the ability of SOD to inhibit the reduction of nitroblue tetrazolium by superoxide. Determination of MDA level was obtained by the method of Varshney and Kale [30]. The CAT activity in the supernatant was measured in accordance with the method of Luck [31]. The GSH-Px activity in the supernatant was measured in accordance with the method of Lawrence and Burk [32]. Reduced GSH was determined according to the method by Beutler [33] using Ellman’s reagent. Protein content of the homogenate and serum were determined using the Biuret method [34].

### 4.6. Statistical Analysis

Data were evaluated for significance by one-way ANOVA. The results are expressed as Mean ± SD of ten rats from each group. The level of statistical significance was set at P < 0.05.

## 5. Conclusions

Ultraviolet spectrophotometer showed that salvianolic acid B, tanshinone II-A or 3,4-dihydroxy-phenyllactic acid (danshensu) were present in *S. miltiorrhiza* ethanol extract 16.8%, 0.84%, 6.2%, respectively. We demonstrate that pretreatment with *S. miltiorrhiza* ethanol extract can prevent I/R-induced myocardial oxidative injury. We supposed that antioxidant activity of SMEE is closely associated with its bioactive chemical components, e.g., salvianolic acid B, tanshinone II-A or 3,4-dihydoxyphenyllactic acid (danshensu).
